# Transgenic Expression of Entire Hepatitis B Virus in Mice Induces Hepatocarcinogenesis Independent of Chronic Liver Injury

**DOI:** 10.1371/journal.pone.0026240

**Published:** 2011-10-12

**Authors:** Bing Na, Zhiming Huang, Qian Wang, Zhongxia Qi, Yongjun Tian, Cheng-Chan Lu, Jingwei Yu, Martha A. Hanes, Sanjay Kakar, Eric J. Huang, J.-H. James Ou, Limin Liu, T. S. Benedict Yen

**Affiliations:** 1 Pathology Service, Veterans Administration Medical Center, San Francisco, California, United States of America; 2 Department of Microbiology and Immunology, University of California, San Francisco, San Francisco, California, United States of America; 3 Department of Laboratory Medicine, University of California, San Francisco, San Francisco, California, United States of America; 4 Department of Molecular Microbiology and Immunology, University of Southern California, Los Angeles, California, United States of America; 5 Department of Pathology, National Cheng Kung University College of Medicine, Tainan, Taiwan; 6 Department of Laboratory Animal Resources, University of Texas Health Science Center, San Antonio, Texas, United States of America; 7 Department of Pathology, University of California, San Francisco, San Francisco, California, United States of America; Saint Louis University, United States of America

## Abstract

Hepatocellular carcinoma (HCC), the third leading cause of cancer deaths worldwide, is most commonly caused by chronic hepatitis B virus (HBV) infection. However, whether HBV plays any direct role in carcinogenesis, other than indirectly causing chronic liver injury by inciting the host immune response, remains unclear. We have established two independent transgenic mouse lines expressing the complete genome of a mutant HBV (“preS2 mutant”) that is found at much higher frequencies in people with HCC than those without. The transgenic mice show evidence of stress in the endoplasmic reticulum (ER) and overexpression of cyclin D1 in hepatocytes. These mice do not show any evidence of chronic liver injury, but by 2 years of age a majority of the male mice develop hepatocellular neoplasms, including HCC. Unexpectedly, we also found a significant increase in hepatocarcinogenesis independent of necroinflammation in a transgenic line expressing the entire wildtype HBV. As in the mutant HBV mice, HCC was found only in aged—2-year-old—mice of the wildtype HBV line. The karyotype in all the three transgenic lines appears normal and none of the integration sites of the HBV transgene in the mice is near an oncogene or tumor suppressor gene. The significant increase of HCC incidence in all the three transgenic lines—expressing either mutant or wildtype HBV—therefore argues strongly that in absence of chronic necroinflammation, HBV can contribute directly to the development of HCC.

## Introduction

Chronic hepatitis B virus (HBV) infection affects around 400 million people worldwide and greatly increases risk of hepatocellular carcinoma (HCC) [1]. The molecular mechanism through which chronic HBV infection contributes to hepatocarcinogenesis remains incompletely understood. Transgenic mice expressing HBV large surface protein that suffer from chronic hepatitis due to different mechanisms eventually develop HCC, suggesting that HBV may be carcinogenic merely by virtue of being an inciter of immune-mediated inflammation and hepatocyte damage [2], [3]. Chronic liver necroinflammation is common in mouse models of spontaneous HCC from targeted deletion of various genes [4], [5], [6], which may or may not closely associated with genetic aberration in human HCC. Besides chronic liver inflammation, HCC is also known to occur in HBV-infected people in the absence of prolonged severe liver injury or cirrhosis [7]; among all people with chronic hepatitis, those with HBV infection had a 7-fold greater risk for HCC than those without HBV infection [8]. Thus although severe hepatitis, of whatever cause, can cause HCC, in natural infections, HBV may play a more direct role in hepatocarcinogenesis. Transgenic mice overexpressing X protein from an HBV subgenomic fragment have been showed to develop HCC [9], but a number of transgenic lines expressing X protein from several constructs do not develop HCC [10], [11]. Importantly, transgenic mice expressing the entire HBV genome have not been shown to increase the incidence of HCC [12], [13], [14], unless the mice are treated with carcinogens [15]. Thus it is unclear if HBV is directly carcinogenic by itself.

**Figure 1:**
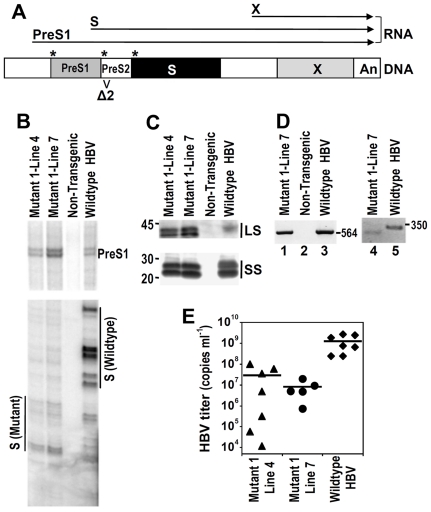
Expression and replication of Mutant 1 HBV in transgenic mice. (**A**) Map of the HBV surface gene, with the 3 initiation codons for the 3 forms of surface protein indicated by asterisks. Also shown are PreS1, S, and X RNA transcripts from HBV. Mutant 1 contains a missense mutation of the preS2 start codon and a 54-bp deletion (marked as Δ2) corresponding to codons 4 through 21 in the preS2 region. (**B**) Primer extension analysis [28] of the preS1 and S transcripts in the liver of Mutant 1 mice, compared to Tg05 wildtype HBV mice. Note that the S transcript products are smaller in the Mutant 1 mice, because of the deletion between the primer and the mRNA start sites [28], and the pattern of start sites is also different from that in wildtype HBV mice, since the deletion extends slightly into the initiation region of the S transcripts[19]. (**C**) Western blotting of the large and small surface proteins in the liver of Mutant 1 and wildtype HBV mice. LS and SS, large and small surface protein respectively. Each protein has 2 forms, differing in the glycosylation. Note that the large surface protein is smaller in the Mutant 1 mice, because of the deletion in the preS2 region. In the top part, the samples were separated on a 10% polyacrylamide gel, while in the bottom part, the samples were separated on a 12% gel. (**D**) PCR detection of circulating HBV [13] in the serum of Mutant 1 mice (lanes 1 and 4), compared to wildtype HBV mice (lanes 3 and 5). For lanes 1-3, the primers (5′ATATTGCCTCTCACATCTCGTCAATCTC and 5′AGCGGTATAAAGGGACTCACGATGCTGT) bracketed nucleotides 101 to 800 in the surface gene, downstream of the deletion. For lanes 4–5, the primers bracketed the deletion in the preS2 region [21]. (**E**) HBV titers in Mutant 1 and wildtype HBV transgenic mice. The amount of HBV genomic DNA in serum of mice at 2-3 months of age is determined by qPCR. The viral titer in wildtype HBV transgenic mice (Tg05) is significantly higher than that in both Mutant 1 Line 4 and Line 7 mice (P<0.02).

The surface proteins constitute the envelope proteins of HBV. Three forms, differing in the initiating codons, are synthesized from a single open-reading frame, with the large form comprising the preS1+preS2+S regions, the middle comprising the preS2+S regions, and the small form having only the S region (Figure 1A). The preS1 promoter-driven transcripts code for the large surface protein, while the S promoter-driven transcripts code for the middle and small surface proteins. The large and small forms are essential for viral morphogenesis and infectivity, but the middle form is dispensable [16], [17]. PreS2 mutants contain in-frame deletions in the N-terminus of the preS2 region as well as a frequent missense mutation of the preS2 start codon. These two mutations result in loss of expression of the middle surface protein and the synthesis of an internally deleted large surface protein and are likely selected because of immune pressure [18]. PreS2 mutants also produce a moderately decreased amount of the small surface protein because the deletion removes a small downstream region of the S promoter [19] (Figure 1A). Recent clinical data have shown that preS2 mutants are found at much higher frequencies in people with HCC than those without [18], [20], [21], and that these mutants are present in apparently clonal nodules of hepatocytes in infected livers [19]. To test if these mutants play a causal role in the genesis of HCC, we generated transgenic mice containing such a mutant HBV genome.

## Materials and Methods

### Transgenic Mice

The BstEII-HpaI fragment of HBV DNA in pSAgΔ2 [19] was used to replace the corresponding HBV fragment in pHBV1.3adw2[14] to generate pHBV1.3adw2Mutant1. The HBV DNA containing 1.3 times of the HBV genome was isolated from the latter plasmid following digestion with PvuII and injected into blastocysts (C57BL/6 X DBA/2 F1) to generate Mutant 1 transgenic mice at the UCSF Comprehensive Cancer Center core facility. The transgenic mice were crossed with B6D2F1/J mice (The Jackson Laboratory, Bar Harbor, ME) and the offspring HBV mice and their litter mates were used in the present study. The wildtype HBV transgenic line Tg05, which had the C57BL/6 genetic background [15], was crossed also with B6D2F1/J mice in the present study. The experimental protocol was approved (AN086079) by the Institutional Animal Care and Use Committee of UCSF.

### Northern and Southern Blotting

The methods were based on Xu et al [14]. Total RNA and genomic DNA was isolated from mouse livers and probed respectively in Northern and Southern blotting with the ^32^P-labled 3.2 kb HBV DNA as described previously [14].

### Primer Extension

Primer extension was conducted with 10 µg of total RNA from mouse liver as described previously [14]. The primer for preS1 transcript was 5′-GGCTCCGAATGCAGGGTCCAACTGATGATCGGG [22], and the primer for S transcript was 5′-AGAGGCAATATTCGGAGCAGGGTTTAC [23].

### Immunoblot and Immunohistochemistry

HBV surface proteins were detected with a goat antibody (DAKO B0560, lot #111) at 1∶3,000 dilution in immunoblot and at 1∶3,500 dilution in immunohistochemistry. Cyclin D1 was detected using a cyclin D1 rabbit antibody (Lab Vision, RM9104) at 1∶1000 dilution in immunoblot and at 1∶100 dilution in immunohistochemistry. An anti-β-catenin antibody (Cell Signaling Technology) was used at 1∶2000 in immunoblot. In all immunoblot, the Amersham ECLPlus kit was used. Immunohistochemical staining was performed on paraffin sections pre-treated by heating in 10 mM sodium citrate at pH 6.0.

### Quantitative PCR (qPCR)

Mouse serum was digested with proteinase K in lysis buffer (20 mM Tris-HCl, 20 mM EDTA, 50 mM NaCl, and 0.5%SDS) at 65°C overnight. HBV viral DNA was then purified by phenol/chloroform extraction and analyzed in real-time quantitative PCR with forward primer (1552-CCGTCTGTGCCTTCTCATCTG-1572), reverse primer (1667-AGTCCTCTTATGTAAGACCTT-1646), and a TaqMan probe derived from nucleotides 1578–1603 (5′-CCGTGTGCACTTCGCTTCACCTCTGC) of the HBV genome. After initial incubation for 2 min at 50°C and 10 min at 95°C, 40 cycles—each consisting of 20 s at 95°C and 1 min at 60°C—of PCR were carried out in an AB-Applied Biosystems machine. A standard curve of HBV DNA was generated for each qPCR experiment, and HBV titers are reported as number of copies per ml of serum.

### Histopathology

Liver samples were fixed with 4% paraformaldehyde in phosphate-buffered saline and embedded in paraffin. Tissue sections were stained with hematoxylin and eosin, and examined blindly by a board certified veterinary pathologist (M. Hanes) and a liver pathologist (T. Yen or S. Kakar).

### Identification of HBV integration loci

Chromosomal integration sites of HBV were identified by a primer extension-dependent PCR [24] using genomic DNA isolated from HBV transgenic mice. A HBV integration site was further confirmed by amplification of a genomic DNA fragment containing one end of the HBV transgene and its flanking mouse sequence through PCR. The following primers are used in amplification by PCR: HBV-I (5′-AGCAAAACAAGCGGCTAGGAGT) and Chromosome 11-I (5′-CTGCTGGGTGACCTGGCTGC) for Mutant 1-Line 7 mice, HBV-II (5′-CAACTGGTGGTTATCATGTATAAAAATGAC) and a primer specific to a LINE1 retrotransposon (5′-ACATAATTGACTACTAGATCCCTGGATG) for Mutant 1-Line 4 mice, and HBV-II and Chromosome 11-II (5′-GGACACATTCATGGAGATTCAGTTTTTC) for Tg05 wildtype HBV mice.

### Metaphase chromosome preparation, karyotyping, and FISH

Lymphocytes were isolated from mouse spleens, cultured in PB-MAX culture medium (Invitrogen) for 72 hours at 37°C, and then incubated overnight with 10 ng/mL Colcemid (Invitrogen). Metaphase cells were harvested and G-banded following a standard mouse cytogenetics protocol [25]. The mouse BAC clone RP23-355K3 corresponding to band E1 of chromosome 11 (11E1; the UCSC genome browser http://genome.ucsc.edu) was obtained from the Children's Hospital Oakland Research Institute. RP23-355K3 and the full-length HBV transgene were labeled with biotin and digoxigenin (DIG) using BioNick DNA Labeling System (Invitrogen) and DIG Nick translation kit (Roche), respectively. These FISH probes were mixed with sheared salmon sperm DNA (Eppendorff) and mouse Cot-1 DNA (Invitrogen), denatured, and then hybridized to the mouse metaphase cells at 37°C overnight. The FISH signals were detected by Avidin-fluorescein (for biotin labeling) or anti-DIG-Rhodamine, and the metaphase chromosomes were counterstained with DAPI II (Abbott Molecular). Metaphase chromosomes and FISH signals were analyzed and documented based on the standard karyotype and nomenclature of mouse [26] using the CytoVision imaging system (Applied Imaging).

## Results and Discussion

### Generation and Characterization of Mutant 1 HBV transgenic mice

To study the effect of a preS2 mutant in transgenic mice, we introduced a 54 base pair deletion (codons 4 through 21) in the preS2 region and a missense mutation of the preS2 start codon into a 1.3X overlength HBV genome that is highly expressed in the liver and produces progeny virions when introduced as a transgene into mice[14]. This mutant (Mutant 1) was found in a patient with HCC[19] and contains the deletion ends most commonly found in a large study[20]. The entire Mutant 1 genome was used for the generation of transgenic mice and two independent lines (lines 4 and 7) were established (Figure 1). Mutant 1 transgenic mice expressed both major classes of HBV transcripts (Figure S1A). Primer extension [27] confirmed that both preS1 and S transcripts were present in the livers of these mice (Figure 1B). Furthermore, although there are background bands in the S primer extension because of strong stops and/or minor start sites, it is clear that, compared to the wildtype HBV transgenic line Tg05, Mutant 1 mice showed a decrease in the relative amounts of S *vs*. preS1 transcripts, as expected from results in transfected cells[19]. Western blotting confirmed the synthesis of a smaller large surface protein than wildtype but a normal-sized small surface protein (Figure 1C). The X protein was expressed in Mutant 1 mice in an amount similar to that in wildtype HBV transgenic mice (Figure S1B). Southern blotting of total undigested liver DNA from Mutant 1 mice showed replicative intermediates of HBV DNA (Figure S1C), albeit at lower levels than wildtype HBV mice, likely because of a decreased activity of the viral polymerase, which is synthesized from an overlapping open-reading frame and hence has a deletion in its non-essential spacer region [1]. The export of viral particles into the serum was confirmed by PCR amplification (Figure 1D, lane 1), while the presence of the preS2 deletion in the genome of these particles was confirmed by using primers bracketing the preS2 region[21], with the serum from Mutant 1 mice giving rise to a band that migrates slightly faster than the band amplified from serum of wild type HBV mice (Figure 1D, lanes 4 and 5, respectively). In addition, we determined HBV titers in the serum of Mutant 1 mice by quantitative PCR (qPCR) (Figure 1E), which are comparable to that in patients with chronic HBV infection [1]. Thus, Mutant 1 mice show appropriate expression and replication of the HBV genome in the liver.

**Figure 2:**
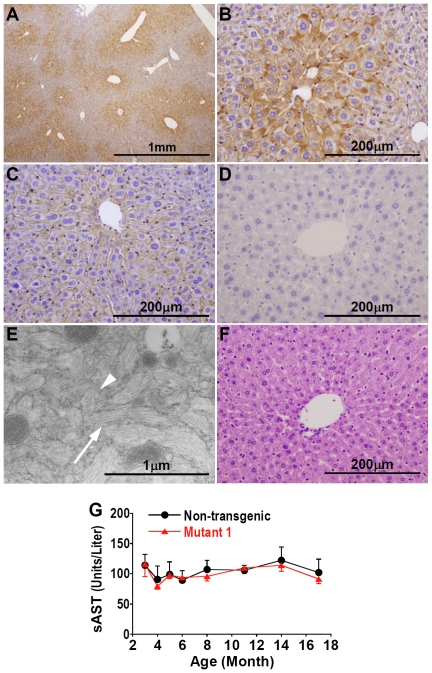
Characterization of younger Mutant 1 mice. (A) Low-power view of liver from 6-month old Mutant 1 mouse stained for HBV surface protein, showing positive cells in a zonal pattern in the liver. (B) High-power view of Mutant 1 liver stained for HBV surface protein, showing strong staining at the periphery of the hepatocyte cytoplasm, identical to the staining pattern of human hepatocytes infected with preS2 mutants [19]. (C) Liver of 6-month old Tg05 wildtype HBV mice stained for HBV surface protein. (D) Liver of non-transgenic mice as a negative control in immunohistochemistry of HBV surface protein. (E) Electron micrograph of a Mutant 1 hepatocyte, showing the presence of long surface protein filaments within the ER (arrow, longitudinal sections; chevron, cross sections). (F) Hematoxylin and eosin stained section of 4-month old Mutant 1 liver, showing the lack of inflammation. (G) Serum AST levels (mean ± SEM) in Mutant 1 mice (12-23 mice for each time point) and non-transgenic littermates (6–11 mice for each time point), showing no significant difference during the first 17 months of age.

The HBV surface proteins are synthesized as transmembrane endoplasmic reticulum (ER) proteins and spontaneously bud into the ER lumen as subviral (genome-free) particles [1]. Small surface protein particles are efficiently secreted, but large surface protein particles cannot be secreted and accumulate in the lumen of the distal ER [28]. Particles composed of mixtures of small and large surface proteins behave in an intermediate manner, with a positive relationship between the percentage of large surface protein and the extent of retention [28]. Because of the relative underexpression of small surface protein by Mutant 1, transfection of this genome into hepatoma cells in culture results in a ∼2-fold decrease in secretion of subviral particles, and patient hepatocytes that harbor mutant HBV show a modest accumulation of surface proteins in hepatocytes[19]. Immunohistochemistry confirmed that surface proteins are expressed at an increased level in a zonal pattern in the liver (Figure 2A) and accumulated in the cytoplasm of hepatocytes (Figure 2B) in Mutant 1 transgenic mice compared to wildtype HBV mice (Figure 2C). There is no staining of HBV surface proteins in non-transgenic mice, indicating the specificity of the assay (Figure 2D). Electron microscopy showed the presence of filaments in the ER, with morphology similar to that from surface protein particles [29] (Figure 2E). High-level expression and retention of HBV large surface protein can lead to cell injury and death[2], [29], [30]. However, possibly because the extent of surface protein accumulation is relatively low in Mutant 1 mice, both histological analysis (Figure 2F) and measurement of serum aspartate aminotransferase (sAST) levels (Figure 2G) showed no evidence of significant hepatocellular injury within the first 17 months of life in Mutant 1 mice. In addition, the livers of Mutant 1 mice had little inflammatory infiltration (Figure 2F). Thus the mice were apparently free from cronic hepatic necroinflammation.

### Hepatocarcinogenesis in HBV transgenic mice

**Figure 3:**
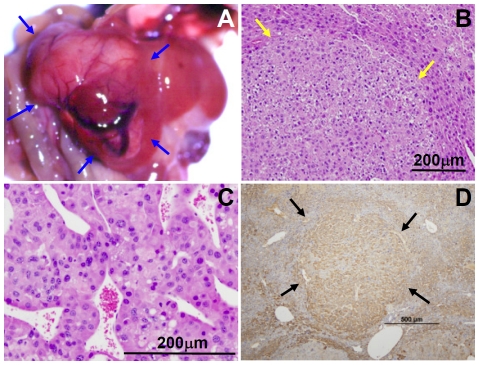
Hepatocellular neoplasms in Mutant 1 mice. (**A**) *In situ* view of a Mutant 1 liver with tumor nodules (arrows). Note the increased vascularity. (**B**) Hematoxylin and eosin stained section of a liver tumor, showing a hepatocellular adenoma (arrows) with mild atypia and compression of surrounding liver parenchyma. (**C**) Hematoxylin and eosin stained section of a liver tumor, showing a HCC with trabecular pattern. (**D**) Immunohistochemical staining for surface protein of a hepatocellular adenoma (arrows) in the liver of a 24-month old Mutant 1 mouse, showing persistent accumulation of surface proteins in the neoplastic cells.

**Figure 4:**
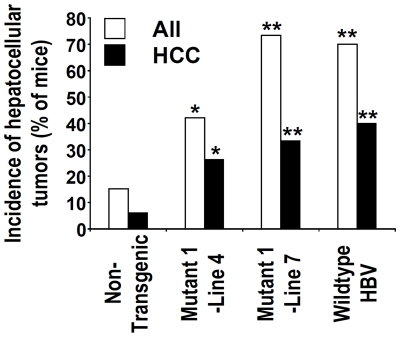
Increase in incidence of hepatocellular tumors in the Mutant 1 and wildtype HBV transgenic mice. Incidence of all hepatocellular tumors (All) and HCC, shown as a percentage of mice with tumor, is from 33 non-transgenic, 19 Mutant 1-Line 4, 30 Mutant 1-Line 7, and 20 Tg05 wildtype HBV male mice. The non-transgenic mice are the littermates of Mutant 1-Line 4, Line 7, or Tg05 mice and because they have comparable tumor incidence, they are grouped together here. Significant differences between the transgenic and non-transgenic mice are indicated by one (P<0.05) or two (P<0.01) asterisks.

Liver tumors were not detected in the Mutant 1 transgenic mice prior to approximately 23 months of age. When the Mutant 1 mice approached 2 years of age, some of them showed abdominal distension. Necropsy revealed that these mice harbored grossly visible liver tumors with increased vascularity (Figure 3A). Microscopic examination confirmed that these tumors represented hepatocellular neoplasms, i.e., hepatocellular adenomas and HCC (Figure 3B and 3C, respectively). While the incidence of hepatocellular tumors in the non-transgenic mice is comparable to previously reported incidence in wildtype non-transgenic mice at 2 years of age [31], [32], the incidence in HCC and all hepatocellular tumors is significantly increased in both lines of Mutant 1 transgenic mice (Figure 4). Similar to the situation in people with chronic HBV infection, HCC development in Mutant 1 mice displays a strong male preponderance (Figure S2). Thus, our Mutant 1 mouse lines represent a transgenic model of HBV carcinogenesis without the use of subgenomic fragments, heterologous promoters, or carcinogens.

**Figure 5:**
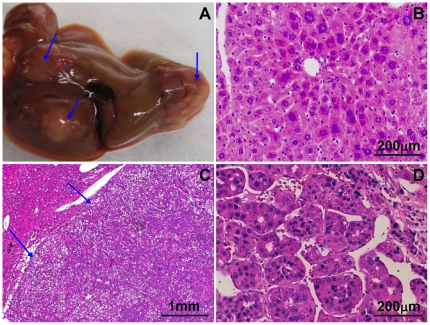
Hepatocellular neoplasms in Tg05 wildtype HBV transgenic mice. (**A**) *In situ* view of a Tg05 liver with tumor nodules (arrows). (**B**) Hematoxylin and eosin stained section of a normal liver in 24-month old Tg05 mouse, showing the lack of inflammation. (**C**) Hematoxylin and eosin stained section of a liver tumor, showing a hepatocellular adenoma (arrows) with mild atypia and compression of surrounding liver parenchyma. (**D**) Hematoxylin and eosin stained section of a liver tumor, showing a HCC with trabecular pattern.

Prompted by our findings that HCC occurs only in aged Mutant 1 transgenic mice, we investigated the possibility that HCC may result also from wildtype HBV when the transgenic mice are aged (Figure 4 and 5). Although HBV titer in Tg05 mice is higher than that in Mutant 1 mice (Figure 1E), Tg05 mice, similar to other wildtype HBV transgenic mice, do not appear to suffer chronic necroinflammation (Figure 5B) [13], [33]. As we reported previously [15], we did not find any hepatocellular tumors in the wildtype HBV transgenic line Tg05 up to 1 year of age (unpublished observation). This is consistent with the findings by Chisari and colleagues that in mouse lines—on C57BL/6 or B10.D2 genetic background—expressing a 1.3X overlength wildtype HBV transgene similar to the one used in Tg05, liver tumor was not detected in animals up to about 1 year of age [13]. Liver tumor or pathological changes were not detected in another wildtype HBV transgenic line of C57BL/6 mice up to 2 years of age [12], but the mouse line expresses a 1.2X overlength HBV transgene which is known to cause a very low level of HBV replication [13]. In contrast to the previous reports, in Tg05 male mice at 2 years of age, both hepatocellular adenomas and HCC were detected (Figure 5). The incidence in HCC and all hepatocellular tumors was significantly higher in the Tg05 mice than in non-transgenic control (Figure 4). It thus appears that age is critical to HCC development in Tg05 mice. The other reasons of the discrepant result of the current study from the previous studies may include HBV expression level and genetic background of the transgenic lines. Importantly, our results strongly suggest that transgenic expression of the full-length wildtype HBV genome in mice can cause HCC.

Although the HBV titer in Mutant 1 mice was lower than that in wildtype HBV mice (Figure 1E), HCC developed with similar penetrance in the Mutant 1 and wildtype HBV mice (Figure 4). The data suggest that compared to wildtype HBV, Mutant 1 might be more oncogenic. This would be consistent with the clinical findings that preS2 HBV variants are significantly associated with HCC [18], [20], [21]. HCC developed in the HBV transgenic mice following long latency. Importantly, the long latency and incomplete penetrance of HCC in our mice mirror the situation in human HBV patients, in whom a minority develops HCC after several decades of chronic infection [34]. Thus our HBV transgenic mice represent appropriate models of human HCC from HBV infection.

#### Mechanistic study of hepatocarcinogenesis in HBV transgenic mice

**Figure 6:**
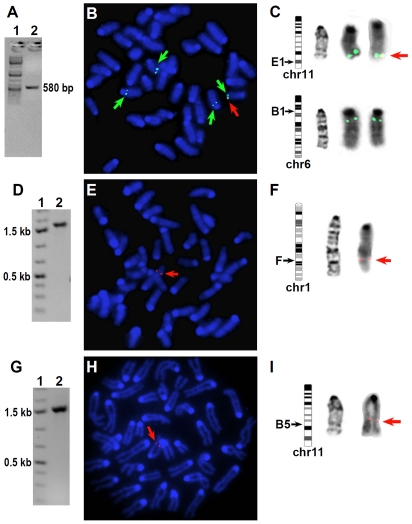
Distinct integration sites of HBV in the mutant 1 and wildtype HBV transgenic mice. The HBV integration sites are identified for Mutant 1-Line 7 (**A–C**), Mutant 1-Line 4 (**D–F**), and wildtype Tg05 (**G**-**I**) mice. (**A**, **D**, and **G**) Detection of an end of the HBV transgene and its flanking mouse sequence by PCR. Lane 1, 1 kb Plus DNA size ladder (Fermentas); lane 2, PCR product. (**B**, **E**, and **H**) Analysis of the integration sites by fluorescent in situ hybridization (FISH). The metaphase cell was stained with an HBV probe (red arrow) and with DAPI (blue). The cell in (**B**) was also stained with a probe (green arrow) derived from the mouse BAC clone RP23-355K3 that corresponds to band E1 of chromosome 11 (11E1). (**C**, **F**, and **I**) Cytogenetic localization of the HBV transgenes. Shown from left to right are the mouse chromosome ideograms, the corresponding G-banded chromosomes, and the DAPI-banded chromosomes with HBV signal from the FISH figures. RP23-355K3 was hybridized to 11qE1 and cross-hybridized to chromosome 6 at band B1. The integrated HBV gene (red arrow) was localized to chromosome 11E1 in Mutant 1-Line 7, to chromosome 1F in Mutant 1-Line 4, and to chromosome 11B5 in wildtype Tg05 mice.

We determined the chromosomal integration sites of HBV in the transgenic mice to see if insertion of the HBV transgenes may affect any oncogene or tumor suppressor gene at the integration sites. By employing a PCR-based method with subsequent DNA sequencing, we found that the HBV transgene was integrated between the Ddx5 (DEAD box polypeptide 5) and Ccdc45 (coiled-coil domain containing 45) genes in chromosome 11qE1 region in Mutant 1 Line-7 mice (Figure 6A and S4). Cytogenetic analyses by fluorescent in situ hybridization (FISH) and Giemsa banding showed that the transgene was integrated into a single position at 11qE1 in Mutant 1 Line-7 mice (Figure 6B and 6C). In Mutant 1 Line-4 mice, the HBV transgene was inserted into the sequence of a LINE1 retrotransposon—one of the most common retrotransposons in the mouse genome—at chromosome 1qF region (Figure 6D-6F and S5). In Tg05 wildtype HBV transgenic mice, the transgene was inserted into a hypothetical open reading frame, LOC66274, at chromosome 11qB5 region (Figure 6G-6I and S6). Thus, the integration sites of HBV are distinct in the three lines of mice and none of the sites is in or next to an oncogene or tumor suppressor gene. The karyotype in all the three transgenic lines appears normal (Figure 6 and data not shown). Therefore HCC in the transgenic mice was unlikely due to chromosomal alterations from HBV DNA integration but rather resulted directly from HBV expression.

Previous transgenic mouse models of HBV-induced HCC, produced by Chisari and colleagues, develop HCC as a result of chronic liver injury and hepatocyte turnover [2], [3]. In contrast, our Mutant 1 and wildtype HBV transgenic mice showed no evidence of chronic liver injury prior to hepatic carcinogenesis (Figure 2F and 2G), and even 2-year old mice did not show significantly increased sAST (Figure S3) or histopathological signs of hepatic necroinflammation (Figure 5B) unless advanced hepatic neoplasms were present, suggesting that liver injury was a consequence of the neoplasm rather than *vice versa*. Furthermore, tumors in Mutant 1 mice showed strong, uniform accumulation of surface proteins (Figure 3D). This finding does not support the notion that accumulation of large surface protein leads to dysfunction or death of these hepatocytes, with compensatory proliferation and subsequent transformation of surrounding healthy hepatocytes that do not express the transgene. Instead, Mutant 1 and wildtype HBV appear to function as cell-autonomous carcinogenic factors and directly promote HCC.

Some [9], [35], but not all [10], [11], lines of transgenic mice containing an HBV DNA fragment with only the X gene spontaneously develop hepatocellular neoplasms, with the overexpression of X protein being the factor determining carcinogenesis. Our Mutant 1 transgenic mice express X protein at a level comparable to that of the wildtype HBV transgenic mice (Figure S1B), which in turn has been reported to be lower than an X-transgenic mouse line that does not spontaneously develop hepatocellular neoplasms[10], [14]. Hepatocarcinogenesis in our transgenic mice thus seems unlikely to result from overexpression of the X protein.

**Figure 7:**
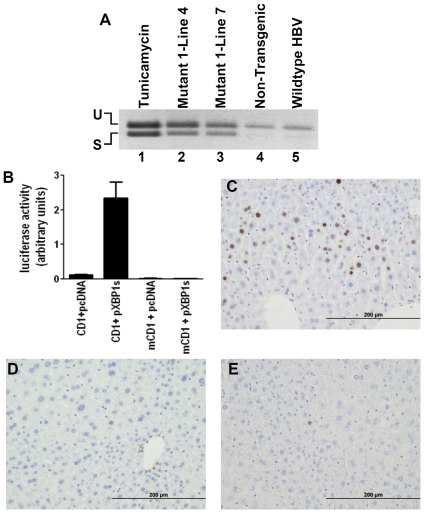
Increase in unfolded protein response (UPR) and cyclin D1 expression in Mutant 1 mice. (**A**) RT-PCR detection of XBP1 mRNA in liver of male Mutant 1, non-transgenic, and wildtype HBV transgenic mice at 4 months of age, using primers that bracket the intron spliced out following UPR activation [47]. The first lane is a positive control from a non-transgenic mouse at 24 hours after injection with 1 mg/kg of tunicamycin. (**B**) Activation of cyclin D1 promoter by XBP1(S) in human hepatoma C3A cells. Reporter constructs with firefly luciferase under the control of either wildtype cyclin D1 (CD1) promoter or a mutant cyclin D1 (mCD1) promoter were cotransfected into C3A cells with the XBP1(S)-expressing pXBP1(S) plasmid [48] or the pCDNA3 vector [49]. The firefly luciferase activity was normalized to *Renilla* luciferase activity from a cytomegalovirus promoter-*Renilla* luciferase plasmid cotransfected into the cells. The data (mean ± SE) are from a representative experiment with triplicates. (**C**–**E**) Immunohistochemical staining for cyclin D1 in liver of half-year-old Mutant 1 (**C**), non-transgenic (**D**), and wildtype HBV transgenic (**E**) mice.

Accumulation of large surface protein in the ER may activate the unfolded protein response (UPR) [36], which occurs in ER stress and is implicated in tumorigenesis [33], [37], [38], [39], [40]. We found that expression and splicing of the mRNA of the UPR-induced transcription factor XBP1 is increased in livers of 4-month-old Mutant 1 mice compared to non-transgenic and wildtype HBV transgenic mice (Figure 7A). The splicing of XBP1 mRNA, which leads to synthesis of the spliced form of XBP1(S), is a specific marker of the UPR [41]. Thus our results indicate activation of UPR by Mutant 1 HBV long before neoplastic transformation. Signaling pathways unrelated to XBP1 have been described in UPR activation [42] and they remain to be explored in Mutant 1 and wildtype HBV transgenic mice. In addition we found that transfection of XBP1(S) in human hepatoma cells activated the cyclin D1 promoter in a reporter assay (Figure 7B). The livers of Mutant 1 mice showed a higher fraction of hepatocytes expressing cyclin D1 than non-transgenic and wildtype HBV transgenic mice (Figure 7C–7E). The increase of Cyclin D1 in Mutant 1 mice unlikely results from hepatocyte death and compensatory regeneration, because hepatocyte proliferation, as indicated by Ki67 staining, is not increased in Mutant 1 mice (Figure S7). Cyclin D1 is important for cell cycle regulation and Cyclin D1 overexpression in hepatocytes of transgenic mice can cause hepatocellular neoplasia [43]. Thus expression of Mutant 1 HBV may promote hepatocellular tumorigenesis in part through activation of UPR and cyclin D1.

**Figure 8:**
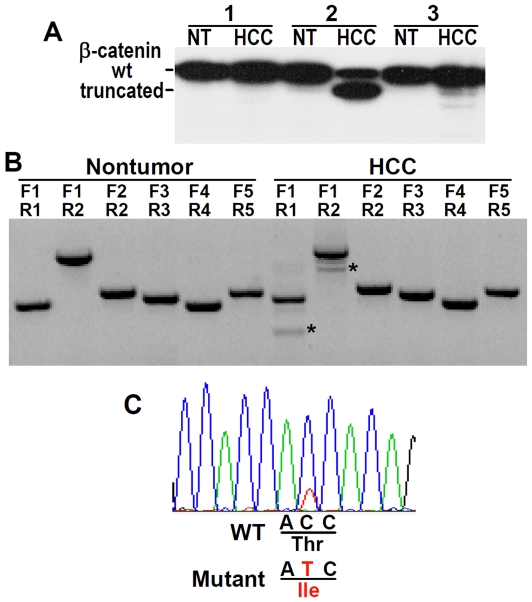
Oncogenic mutation of β-catenin in HCC of Mutant 1 transgenic mice. (**A**) Immunoblot analysis of β-catenin in HCC and associated non-tumor (NT) liver tissues from 3 Mutant 1 trangsgenic mice. One truncated β-catenin was detected in HCC of mouse 2. (**B**) Detection by RT-PCR of the deletion site in the truncated β-catenin shown in (A). The truncated DNA fragments are indicated by asterisks. F1 to F5, forward primers 1 to 5; R1 to R5, reverse primers 1 to 5 [44]. (**C**) Detection by DNA sequencing of T41I, a point mutation in one of the four GSK-3β phosphorylation sites, in HCC from another Mutant 1 transgenic mouse.

β-catenin mutation occurs in both human and mouse HCC and can contribute to tumorigenesis [44], [45]. β-catenin is regulated by casein kinase Iα and glycogen synthase kinase 3β (GSK3β) through phosphorylation of serine and threonine residues in the amino-terminal region encoded by exon 3. Deletion of this region or mutation of the phosphorylation sites results in stabilizing β-catenin in the cytosol while maintaining its signal transduction function in the nucleus [45]. We found, in addition to the 92-kDa full-length β-catenin, a truncated form of β-catenin in one of the fifteen HCC samples examined (Figure 8A). Analysis of the HCC sample by RT-PCR and subsequent DNA sequencing established that the β-catenin mutant missed exactly the entire exon 3 (Figure 8B), which encodes amino acids 5–80 including the phosphorylation sites. We also examined possible point mutations in the phosphorylation sites of β-catenin and found in one HCC sample mutation of Thr-41 (Figure 8C), which would prevent phosphorylation at the site and inactivation of β-catenin by GSK3β [45]. Thus in Mutant 1 HCC, we detected activating mutation of β-catenin at a frequency of 13%, which is close to the incidence of β-catenin mutation in human HCC [46]. In contrast, we did not find any DNA mutation in exons 5–8 of *p53* in 17 HCC samples analyzed, suggesting that p53 mutation may not be involved in the carcinogenesis in Mutant 1 mice.

In summary, we have shown that HBV expression causes no chronic necroinflammation in liver but significantly increases the frequencies of hepatic tumors in three independent transgenic mouse lines. Our findings thus suggest that in addition to causing HCC indirectly by inducing chronic hepatitis and liver injury [2], [3], HBV also has the potential to be directly carcinogenic. The similar latency and penetrance of HCC found in both the Mutant 1 and wildtype HBV mice suggest that Mutant 1 and wildtype HBV may share a common carcinogenic mechanism. Alternatively, Mutant 1 may cause HCC by inducing cyclin D1 expression via UPR whereas other types of stress induced by high level of HBV DNA replication may promote HCC in the wildtype HBV mice. Our transgenic mice provide a clinically relevant model useful for understanding the detailed mechanism of HBV viral carcinogenesis, as well as for exploring whether inflammation, ethanol, and other dietary carcinogens can synergize with HBV in causing HCC.

## Supporting Information

Figure S1 **HBV expression in transgenic mice.** (**A**) Northern blotting of the major HBV transcripts in the liver of Mutant 1 mice and wildtype HBV Tg05mice [1]. The C band corresponds to the precore/core transcripts, while the S band corresponds to the preS1/S transcripts [1]. (**B**) Detection of X protein by Western blotting following immunoprecipitation [2] in the liver of Mutant 1 and wildtype HBV mice. The band marked with an asterisk is non-specific, as shown by its presence in the non-transgenic littermate. (**C**) Southern blotting of HBV replicative intermediates [1] in the liver of Mutant 1 mice and wildtype HBV mice.
(PDF)

Figure S2 **Male preponderance of HCC in Mutant 1 mice.** Shown is the incidence of HCC in Mutant 1 mice (49 male and 15 female) and non-transgenic littermates (33 male and 13 female) at 2 years of age. Significant difference between the male and female Mutant 1 mice is indicated by an asterisk (P<0.05).
(PDF)

Figure S3 **Serum AST levels (mean ± SE) in Mutant 1 mice (n = 24) or non-transgenic littermates (n = 18) at 23–25 months of age, classified by liver histology.** The data in Mutant 1 mice were from 13 mice in the group with no neoplasm, 3 mice in the adenoma group, and 8 mice in the HCC group. P = 0.09, Mutant 1 mice vs. non-transgenic littermates in the non-tumor group.
(PDF)

Figure S4 **Sequences at the junction of HBV (red) and mouse genomic (green) DNA in Mutant 1 Line-7 mice.** The data is derived from sequencing the PCR product shown in Figure 6A. The DNA sequence in green is identical to 106,650,370-106,650,471 of mouse chromosome 11, which is located in 11qE1 region (UCSC Genome Browser).
(PDF)

Figure S5 **Sequences at the junction of HBV (red) and mouse genomic (green) DNA in Mutant 1 Line-4 mice.** The vector sequence is in black. The data is derived from sequencing the PCR product shown in Figure 6D. The DNA sequence in green shares 99.4% identity with 145980892-145980732 of mouse chromosome 1, part of a LINE1 retrotransposon in the 1qF region (UCSC Genome Browser).
(PDF)

Figure S6 **Sequences at the junction of HBV (red) and mouse genomic (green) DNA in Tg05 wildtype HBV transgenic mice.** The vector sequence is in black. The data is derived from sequencing the PCR product shown in Figure 6G. The DNA sequence in green is identical to 78,654,987-78,654,805 of mouse chromosome 11 and is in 11qB5 region (UCSC Genome Browser).
(PDF)

Figure S7 **Immunohistochemical staining for Ki67 in liver of half-year-old non-transgenic (A), Mutant 1 (B), and wildtype HBV transgenic (C) mice.**
(PDF)
