# Supplementary material for: Transgenic Expression of Entire Hepatitis B Virus in Mice Induces Hepatocarcinogenesis Independent of Chronic Liver Injury
Source: PLoS One. 2011 Oct 12;6(10):e26240. doi: 10.1371/journal.pone.0026240 (PMC3192172; doi:10.1371/journal.pone.0026240)
Supplement: Figure S1 — HBV expression in transgenic mice. (A) Northern blotting of the major HBV transcripts in the liver of Mutant 1 mice and wildtype HBV Tg05mice [1]. The C band corresponds to the precore/core transcripts, while the S band corresponds to the preS1/S transcripts [1]. (B) Detection of X protein by Western blotting following immunoprecipitation [2] in the liver of Mutant 1 and wildtype HBV mice. The band marked with an asterisk is non-specific, as shown by its presence in the non-transgenic littermate. (C) Southern blotting of HBV replicative intermediates [1] in the liver of Mutant 1 mice and wildtype HBV mice. (PDF) [file pone.0026240.s001.pdf]

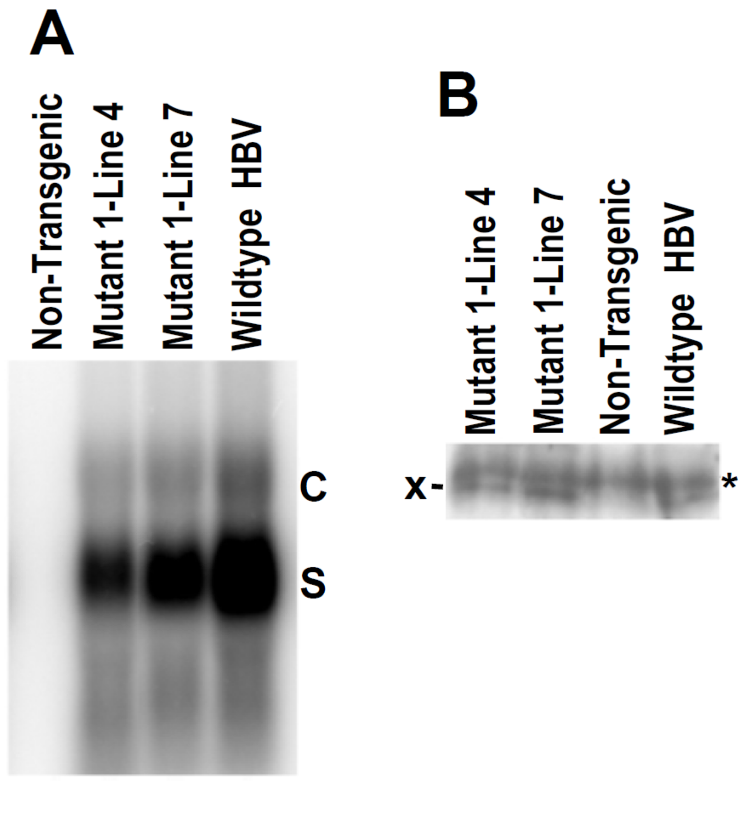

Figure S1. HBV expression in transgenic mice. (**A**) Northern blotting of the major HBV transcripts in the liver of Mutant 1 mice and wildtype HBV Tg05mice [1]. The C band corresponds to the precore/core transcripts, while the S band corresponds to the preS1/S transcripts [1]. (**B**) Detection of X protein by Western blotting following immunoprecipitation [2] in the liver of Mutant 1 and wildtype HBV mice. The band marked with an asterisk is non-specific, as shown by its presence in the non-transgenic littermate. (**C**) Southern blotting of HBV replicative intermediates [1] in the liver of Mutant 1 mice and wildtype HBV mice.

References

1. Xu Z, Yen TS, Wu L, Madden CR, Tan W, et al. (2002) Enhancement of hepatitis B virus replication by its X protein in transgenic mice. J Virol 76: 2579-2584.
2. Lee TH, Finegold MJ, Shen RF, Demayo JL, others a (1990) Hepatitis-B virus transactivator X-protein is not tumorigenic in transgenic mice. J Virol 64: 5939-5947.
