# Supplementary material for: Transgenic Expression of Entire Hepatitis B Virus in Mice Induces Hepatocarcinogenesis Independent of Chronic Liver Injury
Source: PLoS One. 2011 Oct 12;6(10):e26240. doi: 10.1371/journal.pone.0026240 (PMC3192172; doi:10.1371/journal.pone.0026240)
Supplement: Figure S2 — Male preponderance of HCC in Mutant 1 mice. Shown is the incidence of HCC in Mutant 1 mice (49 male and 15 female) and non-transgenic littermates (33 male and 13 female) at 2 years of age. Significant difference between the male and female Mutant 1 mice is indicated by an asterisk (P<0.05). (PDF) [file pone.0026240.s002.pdf]

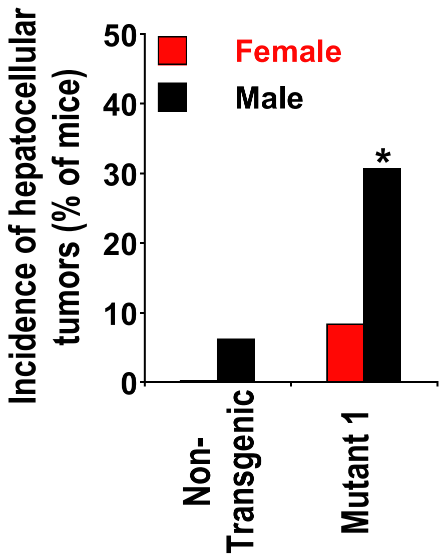

Figure S2. Male preponderance of HCC in Mutant 1 mice. Shown is the incidence of HCC in Mutant 1 mice (49 male and 15 female) and non-transgenic littermates (33 male and 13 female) at 2 years of age. Significant difference between the male and female Mutant 1 mice is indicated by an asterisk ($P < 0.05$).
