# Supplementary material for: Transgenic Expression of Entire Hepatitis B Virus in Mice Induces Hepatocarcinogenesis Independent of Chronic Liver Injury
Source: PLoS One. 2011 Oct 12;6(10):e26240. doi: 10.1371/journal.pone.0026240 (PMC3192172; doi:10.1371/journal.pone.0026240)
Supplement: Figure S3 — Serum AST levels (mean ± SE) in Mutant 1 mice (n = 24) or non-transgenic littermates (n = 18) at 23–25 months of age, classified by liver histology. The data in Mutant 1 mice were from 13 mice in the group with no neoplasm, 3 mice in the adenoma group, and 8 mice in the HCC group. P = 0.09, Mutant 1 mice vs. non-transgenic littermates in the non-tumor group. (PDF) [file pone.0026240.s003.pdf]

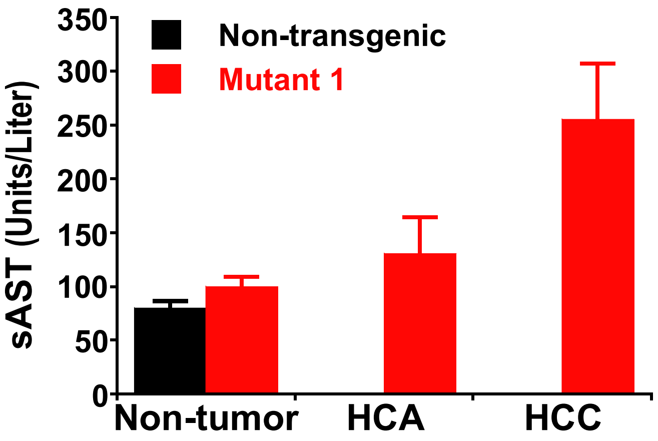

Figure S3. Serum AST levels (mean ± SE) in Mutant 1 mice (n = 24) or non-transgenic littermates (n = 18) at 23-25 months of age, classified by liver histology. The data in Mutant 1 mice were from 13 mice in the group with no neoplasm, 3 mice in the adenoma group, and 8 mice in the HCC group. P = 0.09, Mutant 1 mice vs. non-transgenic littermates in the non-tumor group.
