# Supplementary material for: Transgenic Expression of Entire Hepatitis B Virus in Mice Induces Hepatocarcinogenesis Independent of Chronic Liver Injury
Source: PLoS One. 2011 Oct 12;6(10):e26240. doi: 10.1371/journal.pone.0026240 (PMC3192172; doi:10.1371/journal.pone.0026240)
Supplement: Figure S4 — Sequences at the junction of HBV (red) and mouse genomic (green) DNA in Mutant 1 Line-7 mice. The data is derived from sequencing the PCR product shown in Figure 6A. The DNA sequence in green is identical to 106,650,370-106,650,471 of mouse chromosome 11, which is located in 11qE1 region (UCSC Genome Browser). (PDF) [file pone.0026240.s004.pdf]

5′-CTGCTGGGTGACCTGGCTGCCCTGCCCGCCGATCTGAGAACCCGTAAGCGGGATCTTGACC

GCGGGGAATGGTATCTGGAGCCCGCAAACTGCCGCCTCAGCGGGTATACATTTAAACCCTAACA

AAACAAAAAGATGGGGTTATTCCCTAAACTTCATGGGTTACATAATTGGAAGTTGGGGAACGTT

GCCACAGGATCATATTGTACAAAAGATCAAACACTGTTTTAGAAAACTTCCTGTTAACAGGCCT

ATTGATTGGAAAGTATGTCAAAGAATTGTGGGTCTTTTGGGCTTTGCTGCTCCATTTACACAAT

GTGGATATCCTGCCTTAATGCCTTTGTATGCATGTATACAAGCTAAACAGGCTTTCACTTTCTC

GCCAACTTACAAGGCCTTTCTAAGTAAACAGTACATGAACCTTTACCCCGTTGCTCGGCAACGG

CCTGGTCTGTGCCAAGTGTTTGCTGACGCAACCCCCACTGGCTGGGGCTTGGCCATAGGCCATC

AGCGCATGCGTGGAACCTTTGTGGCTCCTCTGCCGATCCATACTGCGGAACTCCTAGCCGTTGT

TTTTGCT-3′

Figure S4. Sequences at the junction of HBV (red) and mouse genomic (green) DNA in Mutant 1 Line-7 mice. The data is derived from sequencing the PCR product shown in Figure 6A. The DNA sequence in green is identical to 106,650,370-106,650,471 of mouse chromosome 11, which is located in 11qE1 region (UCSC Genome Browser).
