# Supplementary material for: Transgenic Expression of Entire Hepatitis B Virus in Mice Induces Hepatocarcinogenesis Independent of Chronic Liver Injury
Source: PLoS One. 2011 Oct 12;6(10):e26240. doi: 10.1371/journal.pone.0026240 (PMC3192172; doi:10.1371/journal.pone.0026240)
Supplement: Figure S5 — Sequences at the junction of HBV (red) and mouse genomic (green) DNA in Mutant 1 Line-4 mice. The vector sequence is in black. The data is derived from sequencing the PCR product shown in Figure 6D. The DNA sequence in green shares 99.4% identity with 145980892-145980732 of mouse chromosome 1, part of a LINE1 retrotransposon in the 1qF region (UCSC Genome Browser). (PDF) [file pone.0026240.s005.pdf]

5′-ACCCCATTAAAAAACGGGGCTCAGAACTGAACAAAGAATTCTCACCTGAGGAATACCGAAT
GGCAGAGAAGCACCTGAAAAAATGTTCAACATCCTTAATCATCAGGGAAATGCAAATCAAAACA
ACCCTGAGATTCTACCTCACACCAGTCAGAATGGCTAAGGGGGATGTGCTGCAAGGCGATTAAG
TTGGGTAACGCCAGGGTTTTCCCAGTCACGACGTTGTAAAACGACGGCCAGTGCCAAGCTTGCA
TGCCTGCAGGTCGAATCCTGCCTTAATGCCTTTGTATGCATGTATACAAGCTAAACAGGCTTTC
ACTTTCTCGCCAACTTACAAGGCCTTTCTAAGTAAACAGTACATGAACCTTTACCCCGTTGCTC
GGCAACGGCCTGGTCTGTGCCAAGTGTTTGCTGACGCAACCCCCACTGGCTGGGGCTTGGCCAT
AGGCCATCAGCGCATGCGTGGAACCTTTGTGGCTCCTCTGCCGATCCATAC-3′

**Figure S5.** Sequences at the junction of HBV (red) and mouse genomic (green) DNA in Mutant 1 Line-4 mice. The vector sequence is in black. The data is derived from sequencing the PCR product shown in Figure 6D. The DNA sequence in green shares 99.4% identity with 145980892-145980732 of mouse chromosome 1, part of a LINE1 retrotransposon in the 1qF region (UCSC Genome Browser).
