# Supplementary material for: Transgenic Expression of Entire Hepatitis B Virus in Mice Induces Hepatocarcinogenesis Independent of Chronic Liver Injury
Source: PLoS One. 2011 Oct 12;6(10):e26240. doi: 10.1371/journal.pone.0026240 (PMC3192172; doi:10.1371/journal.pone.0026240)
Supplement: Figure S6 — Sequences at the junction of HBV (red) and mouse genomic (green) DNA in Tg05 wildtype HBV transgenic mice. The vector sequence is in black. The data is derived from sequencing the PCR product shown in Figure 6G. The DNA sequence in green is identical to 78,654,987-78,654,805 of mouse chromosome 11 and is in 11qB5 region (UCSC Genome Browser). (PDF) [file pone.0026240.s006.pdf]

5′-TTTTTTTTTTAAAGTAGCCTTTTTCTTTATGTTTTCCTTTTTCTTTTTGTTTATATATTTT

CTGGCCCTGCTTGCTCCGGCCTTATTTATTTTATAAATGTGAGTACACTATTGCTCTTCAGA

TACCAGAAGAGGGCACCAAATCCCATTATAGATGGTTGTGAGCCACCATGTGGTTGCTGGCGAA

AGGGGGATGTGCTGCAAGGCGATTAAGTTGGGTAACGCCAGGGTTTTCCCAGTCACGACGTTGT

AAAACGACGGCCAGTGAATTCGAGCTCGGTACCCATCCTGCCTTAATGCCTTTGTATGCATGTA

TACAAGCTAAACAGGCTTTCACTTTCTCGCCAACTTACAAGGCCTTTCTAAGTAAACAGTACAT

GAACCTTTACCCCGTTGCTCGGCAACGGCCTGGTCTGTGCCAAGTGTTTGCTGACGCAACCCCC

ACTGGCTGGGGCTTGGCCATAGGCCATCAGCGCATGCGTGGAACCTTTGTGGC-3′

Figure S6. Sequences at the junction of HBV (red) and mouse genomic (green) DNA in Tg05 wildtype HBV transgenic mice. The vector sequence is in black. The data is derived from sequencing the PCR product shown in Figure 6G. The DNA sequence in green is identical to 78,654,987-78,654,805 of mouse chromosome 11 and is in 11qB5 region (UCSC Genome Browser).
