# Supplementary material for: Transgenic Expression of Entire Hepatitis B Virus in Mice Induces Hepatocarcinogenesis Independent of Chronic Liver Injury
Source: PLoS One. 2011 Oct 12;6(10):e26240. doi: 10.1371/journal.pone.0026240 (PMC3192172; doi:10.1371/journal.pone.0026240)
Supplement: Figure S7 — Immunohistochemical staining for Ki67 in liver of half-year-old non-transgenic (A), Mutant 1 (B), and wildtype HBV transgenic (C) mice. (PDF) [file pone.0026240.s007.pdf]

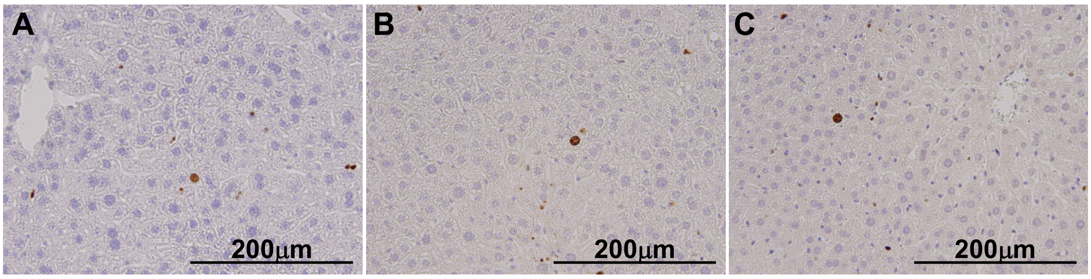

Figure S7. Immunohistochemical staining for Ki67 in liver of half-year-old non-transgenic (**A**), Mutant 1 (**B**), and wildtype HBV transgenic (**C**) mice.
